# Mitochondrial Extracellular Vesicles in CNS Disorders: New Frontiers in Understanding the Neurological Disorders of the Brain

**DOI:** 10.3389/fmolb.2022.840364

**Published:** 2022-03-30

**Authors:** Mary F. Nakamya, Susmita Sil, Shilpa Buch, Ramin M. Hakami

**Affiliations:** ^1^ School of Systems Biology, George Mason University, Manassas, VA, United States; ^2^ Center for Infectious Disease Research, George Mason University, Manassas, VA, United States; ^3^ Department of Pharmacology and Experimental Neuroscience, University of Nebraska Medical Center, Omaha, NE, United States

**Keywords:** mitochondria, mitochondria-derived vesicles, extracellular vesicles, mitophagy, CNS disorders, mitochondrial dysfunction, oxidative stress

## Abstract

Recent findings have highlighted potential diagnostic and prognostic values of extracellular vesicles (EVs) that contain mitochondrial derived components for neurological disorders. Furthermore, functional influences of vesicles carrying mitochondrial components have been reported. In particular, this includes indications of crosstalk with mitophagy to influence progression of various CNS disorders. In this mini-review, we discuss the current state of knowledge about this intriguing class of vesicles in neurological disorders of the CNS, and outline the lacunae and thus scope of further development in this fascinating field of study.

## Introduction

During the past decade, investigations into the CNS functions of extracellular vesicles (EVs) has opened a new window into our mechanistic understanding of the neurological disorders of the brain. However, much still remains to be deciphered and explained. More recently, functional studies of vesicles that contain mitochondrial components, which include EVs, have expanded this new frontier of understanding and added to the excitement surrounding EV studies. In this mini-review, we provide a synthesis of what is currently known about vesicles that carry mitochondrial cargo, with a focus on several neurological disorders of the CNS. As part of this, we will also discuss current findings that highlight cross talk between mitophagy and the EVs that carry mitochondrial cargo. At the end of the review, we identify several existing gaps of knowledge and suggest fruitful future directions to pursue.

### EV Properties and Subpopulations

EVs are secreted membrane-enclosed “packages” that carry various components including proteins, RNA and DNA (Colombo et al., 2014; Fleming et al., 2014; Takahashi et al., 2017). EVs can directly transfer bioactive material between cells, initiate signaling events at the cell surface (Colombo et al., 2014; Olanrewaju and Hakami, 2020), regulate the molecular composition of the extracellular milieu (Iraci et al., 2017), and be involved in cellular homeostasis of EV-secreting cells (Melentijevic et al., 2017; Takahashi et al., 2017). Based on mechanisms of biogenesis, release pathways, size, and content, EVs are grouped into three main types; microvesicles (MVs), exosomes, and apoptotic bodies (Raposo and Stoorvogel, 2013). Exosomes are approximately 30–150 nm in diameter and are derived from the endosomal pathway, while microvesicles (MVs) or ectosomes are formed directly through plasma membrane budding. Apoptotic bodies, which are typically >1,000 nm in diameter are formed by cells that are undergoing cell death (Battistelli and Falcieri, 2020). EVs can cross the blood-brain-barrier (BBB) easily, facilitating material exchange between the CNS and blood circulation (Banks et al., 2020). They carry messages involved in the physiology of CNS, such as regulation of neuronal firing, synaptic plasticity, and myelin formation (Bátiz et al., 2015; Gassama and Favereaux, 2021). Upon crossing the BBB, EVs can regulate neuroinflammation that results in systemic pathology of the CNS (Upadhya and Shetty, 2019; Upadhya and Shetty, 2021). EVs also influence the aggregation and clearance of toxic proteins in neurodegenerative diseases (Holm et al., 2018).

The differentiation of whether the CNS effects are mediated by exosomes or MVs can be challenging due to their close biophysical characteristics and overlapping molecular markers (Phinney et al., 2015; Hurwitz et al., 2016). Although there are guidelines proposed to aid in the identification of different EV subpopulations (Théry et al., 2018), the specific criteria for characterization of the mitochondria-derived vesicles (MDV) is still lacking. The guidelines propose that EVs can be broadly sub-divided based on their physical and biochemical characteristics and conditions/sources of their production. Accordingly, categorization of EVs has been proposed based on the following parameters: 1) Centrifugation speed - EVs derived from medium speed centrifugation can be categorized as oncosomes (Minciacchi et al., 2015), ectosomes (Keerthikumar et al., 2015; Haraszti et al., 2016), microvesicles (Haraszti et al., 2016), cell debris (Keerthikumar et al., 2015), or large/medium vesicles (Kowal et al., 2016; Durcin et al., 2017), whereas EVs that are obtained by ultracentrifugation at 100,000 xg are commonly referred to as exosomes; 2) Specific protein markers- The presence of proteins such as transmembrane or GPI-anchored proteins indicate the lipid bilayer structure specificity of EVs, and also whether these EVs bud off directly from the plasma membrane or following transit through the endosomal pathway. The presence of cytosolic proteins (eukaryotic cells and Gram-positive bacteria) or periplasmic proteins (Gram-negative bacteria) demonstrate that the analyzed preparation displays the structure of lipid bilayers enclosing the intracellular material; 3) Phospholipid content of lipid bilayers - Although proteins are emphasized in the literature as the key markers to differentiate between various EV subtypes, the nature of phospholipids present in the EV lipid bilayer can also serve as markers for differentiating EV sub-populations (Record et al., 2014; Skotland et al., 2017). However, the ratios of various lipids such as cholesterol, sphingomyelin, ceramide, and phosphatidyl-choline/ethanolamine/inositol in the EVs, and how these ratios differ in sub-populations, have not yet been established, and additional comparative lipidomic studies of separated EVs and lipoprotein subtypes are warranted in the field (Théry et al., 2018); 4) Functional properties of EVs- Based on their functionality, EVs can be further categorized, comparing quantitatively the effects of EV fraction(s), EV-depleted fraction(s), and the initial unfractionated fluid, to identify the relative contributions of each to total activity; 5) Origination source of EVs - Identifying the source of EV production is another means of categorizing EVs. For example, mesenchymal stromal cell (MSC) EVs function differently than milk EVs, urine EVs, or cancer cell EVs. As MISEV 2018 recommends, for analysis of certain EV-associated functions, the topology of EV-associated components may also need to be assessed; in other words, whether a component is luminal or on/at the surface of EVs. A determination of topology could be particularly important for certain classes of biomolecules. Protease and nuclease digestions, detergent permeabilization, and targeting antibodies to outer epitopes (should bind) or inner epitopes (should not bind) could be used to probe topology.

### Mitochondria and MDVs

Mitochondria are double membraned organelles that regulate several physiological processes, such as calcium signaling, apoptosis, and cell metabolism (Friedman and Nunnari, 2014; Gustafsson et al., 2016). The mitochondrial genome may exhibit homoplasmy or heteroplasmy and the latter can be either maternally inherited or arise from somatic mutations that occur during tissue development and aging (Taylor and Turnbull, 2005). Somatic mutations in mitochondrial DNA (mtDNA) are associated with pathologies such as cancer, cardiovascular diseases, neurodegenerative disorders, and aging (Wallace, 2013). The integrity of mitochondrial genome is controlled by continuous cycles of fusion and fission that distribute mitochondrial proteins, lipids, and DNA between these cell organelles (Mishra and Chan, 2014). MtDNA can behave as damage-associated molecular patterns (DAMPs) and activate an innate immune inflammatory response in macrophages or neutrophils (Nakahira et al., 2015), and neuroinflammation in the CNS (Madsen et al., 2017). In addition, mitochondria produce reactive oxygen species (ROS) via the electron transport chain during ATP production and are also susceptible to it (Barja, 2013). When mitochondrial homeostasis is severely compromised, for example due to an imbalance in the cellular redox system, mitochondrial permeability transition pore (mPTP) opens to transfer mitochondrial components, including mtDNA, into the cytosol to trigger mitochondrial fission and mitophagy that clears damaged mitochondria from the cell (Picca et al., 2020a). Different studies have shown that multiple cell types also secrete mitochondrial components into the culture media (Puhm et al., 2019). Interestingly, entire mitochondria can also be transferred between cells (Hayakawa et al., 2016). Mitochondrial stress stimulates the release of specific molecules, including mtDNA and DAMPs, that have strong proinflammatory activity (Sliter et al., 2018). Furthermore, slightly damaged mitochondria and other mitochondrial constituents can be shuttled outside the cell via EVs, in effect outsourcing mitophagy in addition to allowing intercellular signaling crosstalk (Phinney et al., 2015; Hayakawa et al., 2016). For instance, in stem cells, EVs translocate mitochondrial genes that impact cellular functions in the receiving cells, such as translation, differentiation, cellular reprogramming, and inflammatory signaling pathway activation (Phinney et al., 2015). Therefore, studies that can deconvolute the relationship between mitochondrial hemostasis and mitophagy hold the promise of discovering novel therapeutic targets for treatment of neurodegenerative diseases.

Mitochondria-derived vesicles (MDVs) are small vesicles with a diameter of 70–150 nm that are known to facilitate communication between mitochondria and other organelles (Sugiura et al., 2014). MDVs can originate either from the outer membrane or from both the outer and inner membranes of mitochondria (Neuspiel et al., 2008). They can carry specific mitochondrial contents to the late endosome/multivesicular bodies for packaging into EVs (Soubannier et al., 2012b; Pérez-Treviño et al., 2020). Alternatively, MDVs containing oxidized mitochondrial cargo can be transported to lysosomes for degradation (Pérez-Treviño et al., 2020; Riley and Tait, 2020). Furthermore, MDVs carrying mitochondrial-anchored protein ligase (MAPL) are transported to peroxisomes (Neuspiel et al., 2008). Using confocal and electron microscopy studies and analyzing various cell types, Soubannier et al. have shown that during stressed-induced conditions there is increased levels of transport of MDVs carrying oxidized cargo to lysosomes for degradation, and that this process occurs without triggering mitophagy (Soubannier et al., 2012a). For instance, ATG^+/+^ and ATG5^−/−^ mouse embryonic fibroblasts (MEFs) transfected with the mitochondria matrix marker OCT-DsRed, and infected with a dominant negative mutant of DRP1 to block mitophagy [DRP1 (K38E)], generated the same numbers of MDVs following treatment with stress-inducing glucose oxidase (GO). Also, Tom20+ (outer mitochondrial membrane protein) or PDH+ (matrix enzyme) MDVs that were generated within stressed COS7 cells grown on galactose (to induce mitochondrial respiration) and in the presence of DRP1(K38E), lacked LC3 protein (Soubannier et al., 2012a). Therefore, MDV formation and trafficking of their oxidized cargo to lysosomes is distinct from DRP1-dependent mitophagy. Consistent with these findings, Caielli et al. have reported that neutrophils show steady state production of mtDNA that is positive for 8-hydroxy-2-deoxyguanosine (8OHdG), a marker of DNA oxidation, and is sorted to lysosomes based on colocalization with LAMP(+) compartments in the presence of Bafilomycin-A (Caielli et al., 2016). The authors suggest that this sorting mechanism could likely be mediated by MDVs. Furthermore, the analysis of 8OHdG (+) vesicles demonstrated that similar to the lysosome-targeting MDVs reported by Soubannier et al. these vesicles also contain the IMM proteins, PDH, and mtDNA (Caielli et al., 2016).

The mtDNA released from damaged mitochondria, which can be packaged into MDVs carrying cargo destined for extracellular release, can stimulate pro-inflammatory pathways through interaction with TLR and the Stimulator of Interferon Genes (STING) that lead to activation of NFκB and type I Interferon (Picca et al., 2018). However, following mitochondrial stress, stabilization of STING through interaction with Tollip may be compromised, resulting in a dampening of the innate immune response (Ryan and Tumbarello, 2021). Collectively, these results indicate that MDV formation or trafficking must be regulated during pathological conditions, particularly those with high dependence on mitochondrial function such as CNS diseases. To understand these regulatory processes, isolation and characterization of various MDV subtypes and the EVs that derive from them are important to achieve. Recently, using a novel high-resolution density gradient method to isolate brain EVs, D’Acunzo et al. achieved purification of a new subtype of EVs that carry multiple mitochondrial proteins and were named mitovesicles (D’Acunzo et al., 2021). Mitovesicles differ by morphology and content from MDVs (D’Acunzo et al., 2021), and further investigations of their functions and utility should provide important information. In the following sections, we will provide a synthesis of the current knowledge of contributions of MDVs and EVs carrying mitochondrial cargo to various CNS disorders and the implications of these findings.

### Parkinson’s Disease

Mitochondrial dysfunction (MD) is associated with neurodegenerative illnesses, including Parkinson’s disease (PD) (Chen et al., 2019). PD symptoms include motor impairments such as resting tremor, bradykinesia, rigidity, postural instability, and non-motor symptoms such as sleep perturbations, constipation, cognitive impairment, and depression (Alexander, 2004; Krüger et al., 2017). Progressive damage of dopaminergic neurons of the substantia nigra pars compacta and dopamine exhaustion in the striatum is the chief cause of PD (Alexander, 2004). In PD, MD can impair mitochondrial biogenesis, increase ROS production, compromise trafficking of cellular material, impair electron transport chain (ETC) function, alter mitochondrial dynamics, cause calcium imbalance, and disrupt mitophagy (Picca et al., 2020a). Therefore, cells tightly regulate mitochondrial homeostasis through processes such as mitophagy and MDVs that are functionally linked together. Mitophagy is initiated by the depolarization of the mitochondrial membrane via the PINK1–Parkin pathway (Whitworth and Pallanck, 2017), the mutations in which are responsible for the early onset recessive form of PD. PINK1 is a mitochondrially targeted kinase and Parkin serves as a cytosolic ubiquitin ligase. Mitochondria import and degrade PINK1 under normal conditions (Matsuda et al., 2010; Narendra et al., 2010); however, during MD, its import is halted and PINK1 accumulates on the outer membrane of the mitochondria and attracts Parkin to the mitochondrial surface (Narendra et al., 2008) via the phospho-ubiquitin chains (Kondapalli et al., 2012). PINK1 then phosphorylates Parkin and repeated phosphorylation-ubiquitination reactions promote ubiquitination of many outer membrane proteins (Pickrell and Youle, 2015), which are engulfed by autophagosomes for mitophagy (Pickrell and Youle, 2015; Yamano et al., 2016). Although the exact mechanisms that lead to MDV biogenesis is unclear, PINK1 and Parkin regulate this process as well, providing a mechanistic link between mitophagy and MDVs (Sugiura et al., 2014). PINK1 and Parkin can repress MDV formation by inhibiting the recruitment of Rab9 and Sorting nexin 9 to mitochondria, hence inhibiting a mitochondrial antigen presentation pathway that relies on MDVs instead of mitophagy and preventing the ensuing inflammatory responses that contribute to PD pathology (Matheoud et al., 2016).

In mammalian cells, unbalanced fission leads to mitochondrial fragmentation (Chan, 2006). Increased mitochondria fragmentation results in high levels of circulating cell free-DNA (ccf-DNA) that increases inflammation, and is regulated by Dynamin-like protein 1 (DLP1) and Fis1 (Chan, 2006). DLP1 is a cytosolic protein that is localized to the mitochondrial outer-membrane, which are sites of fission activity (Smirnova et al., 2001). The mitochondrial DLP1 complex turnover is mediated by VPS35, a key component of the retromer complex, and its dysregulation causes MD that is critical to PD pathogenesis (Chan, 2006). VPS35 is also linked to the production of MDVs (Hanss et al., 2021), which can traffic cargo from mitochondria to peroxisome or lysosome (Sugiura et al., 2014). Wang et al. demonstrated that the retromer complex interacts with VPS35-DLP1 and mediates the removal of DLP1 complexes from mitochondria via trafficking of MDVs to lysosomes, hence allowing for efficient mitochondrial fission (Wang et al., 2016). They also showed that PD-associated VPS35 mutations in persons with sporadic PD results in an elevated VPS35-DLP1 interaction that improves the retromer-dependent turnover of mitochondrial DLP1 complexes via MDV trafficking, leading to excessive fission and therefore MD (Wang et al., 2016). Additionally, increased ROS augments the VPS35-DLP1 interaction, which could explain the improved VPS35-DLP1 interaction seen in the sporadic PD brains (Wang et al., 2016). In summary, accumulated evidence shows the critical role of MDVs that involves mitophagy crosstalk in regulation of mitochondrial dynamics and quality control that are essential for PD progression and pathogenesis. It should also be noted that analyzing the cargo of circulating small EVs (sEVs)/exosomes, which can function to release mitochondrial components into circulation, could also contribute to a deeper mechanistic understanding of PD, as well as help identify candidate PD biomarkers. The EXosomes in PArkiNson disease (EXPAND) study has addressed this by characterizing the cargo of sEVs/exosomes from sera of PD patients and comparing them with those from healthy individuals (Picca et al., 2019; Picca et al., 2020b).

### Down Syndrome

The Down syndrome (DS) is the most common aneuploidy and cause of intellectual inability of genetic origin, and exhibits Alzheimer’s disease (AD) pathology at a young age (Capone et al., 2018). Presence of a third copy of a portion of chromosome 21 (Hsa21) in DS results in overexpression of several genes, including the amyloid precursor protein (APP), which causes AD pathologies such as neuroinflammation, neuronal cell loss, amyloid plaques, and neurofibrillary tangles (NFTs) (Hartley et al., 2015; Delabar et al., 2016). DS is associated with the absence of mitophagy, causing the buildup of toxic mitochondrial components in the brain cells (Bordi et al., 2019) and consequently inflammation and neurodegeneration. Using both normal and DS human fibroblasts, Bordi et al. showed that macroautophagy, mitophagy, and the clearance of damaged mitochondria by mitophagy, can be rescued by inhibiting mTORC1 and mTORC2, suggesting a possible strategy for treatment of DS (Bordi et al., 2019). In addition, mutations in mtDNA and impaired mtDNA repair systems have been reported in fibroblasts within DS brain tissues (Druzhyna et al., 1998; Coskun and Busciglio, 2012).

It has been shown that in trisomic mouse model Ts [Rb (12.1716)]2Cje (Ts2) (Villar et al., 2005) the levels of brain-derived EVs named mitovesicles are increased in brains from Ts2 compared to diploid (2N) littermates (D’Acunzo et al., 2021). The levels of proteins such as cytochrome c oxidase subunit 4 (COX-IV) and the pyruvate dehydrogenase E1 component subunit alpha (PDH-E1α), associated with mitochondrial matrix, are increased in the Ts2 brain mitovesicles, as well as in the *in vitro* mitochondrial stress model produced by treatment with antimycin-A, a mitochondrial respiratory-chain inhibitor that leads to production and accumulation of ROS without inducing mitophagy (D’Acunzo et al., 2021). These results suggest a dysfunction in oxidative phosphorylation similar to that observed in DS, and provide evidence that mitovesicles may serve as a biomarker to evaluate brain MD in neurological disorders. Related to this, during oxidative stress, brain cells increase the transfer of damaged mitochondria encapsulated into EVs from astrocytes to neurons (Hayakawa et al., 2016). Although mitovesicle levels are increased in DS brains, their composition shows reduced levels of proteins involved in ATP production such as UQCRC2 and SDH-B, suggesting impaired ATP synthesis (D’Acunzo et al., 2021). Increased levels of mitovesicles, which lack external membranes, in DS brains could be in response to ATP demands to counteract the oxidative phosphorylation impairment (D’Acunzo et al., 2021). In support of this, deficiency of proteins involved in ATP production such as ATP synthase and ADP/ATP translocator were reported in human skin fibroblasts with trisomic karyotype under mitochondrial stress (Valenti et al., 2010, Valenti et al., 2011). As it was mentioned earlier, DS is associated with lack of mitophagy, which causes accumulation of damage-associated molecular patterns (DAMPs) due to mitochondrial stress and can lead to an inflammatory response by innate immunity (West et al., 2011; Mills et al., 2017). Although the mechanisms of release of DAMPs into the extracellular space is not clearly known, it was recently shown that the cellular decision of whether damaged mitochondrial content is packaged into EVs is dependent on selective targeting to one of two distinct MDV pathways (Todkar et al., 2021). Specifically, mitochondrial proteins are packaged into the inner membrane/matrix MDVs whose formation depends on the function of Optic Atrophy 1 (OPA1) and sorting nexin 9 (Snx9) (Todkar et al., 2021). Further studies are required to investigate whether MDVs are intensifying the disease or are extracting damaged mitochondrial components to restore the redox system in DS brains.

### Alzheimer’s Disease

Alzheimer’s disease (AD) is a neurodegenerative disease, with dementia-like symptoms. There are two forms of Alzheimer disease (AD); a late-onset sporadic form (SAD) and an early-onset familial form (FAD). AD is characterized by amyloid β (Aβ) plaques, tau-containing neurofibrillary tangles, and neuronal inflammation/loss leading to brain atrophy (Weller and Budson, 2018; DeTure and Dickson, 2019). The toxicity associated with Aβ aggregates can trigger mitochondria damage, causing oxidative injury and consequently MD in AD (Kim et al., 2020). EVs isolated from astrocytes, microglia, and neurons exposed to Aβ aggregates and H_2_O_2_ show the presence of mitochondrial structures and mitochondrial RNA (mt-RNA) and proteins (Kim et al., 2020), supporting the role of MD in AD and indicating MDV involvement. One interpretation of these findings is that EVs export toxic mitochondrial components from damaged mitochondria, contributing to cellular pathologies and AD.

Current diagnosis of AD relies on biomarkers obtained through invasive procedures to obtain cerebrospinal fluid (CSF) (Jack et al., 2016). Furthermore, the diagnosis is limited to the adverse stages of the disease, which complicates treatment regimens. Therefore, a search for novel diagnostic markers and therapeutic strategies is warranted. EVs have shown great promise for use as AD biomarkers (Kapogiannis et al., 2019; Pulliam et al., 2019; Kim et al., 2020). Villar-Vesga et al. characterized systemic EVs from postmortem samples of sporadic Alzheimer’s disease (SAD) and Familial Alzheimer’s disease (FAD) patients, and observed increased levels and size of systemic EVs in both groups of patients (Villar-Vesga et al., 2020). They also found that SAD patients showed an increase in endothelial- and leukocyte-derived EVs containing mitochondrial markers whereas FAD patients showed an increase in platelet-derived EVs. The increased expression of the mitochondrial markers such as DIOC6 in SAD-EVs could be due to high levels of mitochondrial components from dysfunctional mitochondria (Villar-Vesga et al., 2020). Furthermore, using RNA-Seq analysis, Kim et al. (2020) reported increased mitochondrial (mt)-RNAs, such as MT-ND1-6 mRNAs and other protein-coding and non-coding mt-RNAs, in plasma EVs of mild cognitive impairment (MCI) and AD individuals as compared with healthy controls. EV contents such as proteins, mRNAs, and microRNAs can serve as diagnostic or prognostic biomarkers in various pathologies such as cancer, kidney disease, and cardiovascular disease (Dickhout and Koenen, 2018; Ståhl et al., 2019). In line with this, the studies discussed here highlight the potential of EV-based biomarkers in plasma for diagnostic and/or prognostic studies of MCI and AD.

### Aging

Aging occurs due to the progressive decline of biological functions and failure of the organism’s ability to adapt to metabolic stress over time, increasing the risk of various adverse health conditions and diseases (Cesari et al., 2016). Studies to elucidate the process of aging are vital for developing strategies that can delay its onset and prevent associated diseases. Proposed causes of aging include loss of proteostasis, genomic instability, telomere attrition, deregulated nutrient sensing, epigenetic alterations, altered intercellular communication, stem cell exhaustion, cellular senescence, and MD (López-Otín et al., 2013). During aging, impairment of autophagy and other cellular-degradation mechanisms in removing damaged cytosolic materials such as dysfunctional mitochondria disrupts cellular homeostasis and leads to accumulation of intracellular “waste” (Terman et al., 2010; Yin et al., 2016). MD has long been connected to the aging process and related diseases (Park and Larsson, 2011; Ross et al., 2013). In this connection, mutations of mtDNA cause early aging in murine models (Park and Larsson, 2011) and increase with human age (Zhang et al., 2017). In peripheral blood mononuclear cells, mtDNA copy numbers reduce with human age (Mengel-From et al., 2014). Also, ccf-mtDNA from cellular damage or stress increase progressively past the age of 50 (Pinti et al., 2014; Picca et al., 2017). A recent European study has shown a slight decline in ccf-mtDNA levels comparing children to middle-aged individuals, followed by a gradual increase in the elderly (Pinti et al., 2014). Consistent with this study, Lazo et al. reported that ccf-mtDNA in plasma EVs decreased with human age in both cross-sectional and longitudinal studies of a middle-aged cohort (∼30–69 years) (Lazo et al., 2021). Also, maximal mitochondrial respiration of cultured cells exposed to EVs from old donors versus EVs from young donors showed differential affects, suggesting that EVs affect mitochondrial energetics in an age-dependent manner (Lazo et al., 2021). Together, the data suggest that EV-associated ccf-mtDNA may indicate and/or contribute to various physiological and pathological conditions related to aging, and that age-dependent packaging of EVs (which might also affect ccf-mtDNA levels) could play an important role in these processes.

## Conclusion and Future Perspective

**FIGURE 1:**
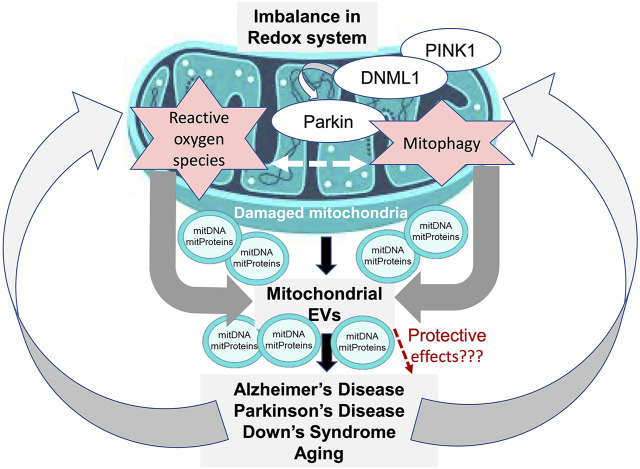
Model of mitochondrial EV involvement in CNS disorders. Dysregulated mitophagy and induction of oxidative stress are associated with mitochondrial dysfunction (MD), which leads to generation of mitochondria-derived vesicles (MDVs) that contain cargo from the damaged mitochondria, including mitochondrial DNA (mitDNA) and mitochondrial proteins (mitProteins). The MDV cargo can be transferred to vesicles that are released extracellularly as a part of the disease pathogenesis and lead to further disease progression. Alternatively, the MDVs may contribute to a protective mechanism by being shuttled to lysosomes for destruction of their cargo.

There is a growing interest in understanding the physiological effects and utility of the mitochondrial cargo that gets packaged into EVs following mitochondrial damage during various CNS disorders (Figure 1). The ability to separate and characterize mitovesicles as a new brain-derived EV subtype, which has been recently achieved as part of DS studies, ushers in a new area of investigation. Future work on understanding the various CNS functions of mitovesicles and how they influence CNS pathologies other than DS should prove highly rewarding, and as part of this effort the purification and analysis of mitovesicles from other tissue types will be very informative as well. Such studies will enable a deeper understanding of the specific roles of EV subtypes in the CNS, as carriers of either neuroprotective, anti-inflammatory, or proinflammatory cargos. As an intriguing possibility, this knowledge might allow development of approaches to alter EV cargo in order to limit inflammation, improve regeneration, or halt degeneration in target cells.

As we have discussed here, mitochondrial EVs have a strong potential for use as biomarkers or development of therapeutic interventions for CNS disorders. However, given their heterogeneity, there is still a need for significant additional basic research into their various functions, and also technical advances to isolate and study the various EV subtypes, before they could be applied for detection or treatment of CNS diseases. One fascinating and seldom-studied aspect for future investigation is the role of the lipid cargo in EVs, using both *in vitro* and *in vivo* systems. In addition, although redox imbalance has been implicated as a trigger of mitochondrial EV production *in vitro*, other mechanisms governing their production and release *in vitro* and *in vivo* remain to be investigated. Another important aspect to be delineated in future work is the functional similarities and differences that exist between MDVs, which directly derive from the mitochondrial membrane, and EVs that carry mitochondrial components.

Although some limited studies related to mitochondrial EVs have been performed for several CNS disorders, there is even less work done in this area for additional CNS pathologies. For instance, it has been shown that EVs extracted from serum of children with autism spectrum disorder (ASD) contain higher protein content compared to normal children and that these EVs stimulate cultured human microglia to secrete significantly more IL-1β as compared to the control, probably due to high levels of mtDNA (mtDNA7S) (Tsilioni and Theoharides, 2018). However, so far, this is the only study of these EVs and much further work is needed to develop an in-depth knowledge of their functions during ASD. Similar to the ASD findings, analysis of EVs extracted from serum of subjects with non-penetrating traumatic brain injury (TBI) showed a significant increase in mtDNA in TBI patients compared to the control group (Marcatti et al., 2021), and this also needs to be followed up with extensive functional studies.

While the current information on the role of EVs and MDVs in CNS disorders is still very limited, it is nevertheless highly intriguing and highlights exciting areas of investigation that remain to be explored. We strongly believe that the next decade will witness an explosion of research in the field of mitochondrial EVs and their roles in disease pathogenesis.
